# Association between frailty and incidence of AMD among middle-aged and older people: evidence from the UK Biobank

**DOI:** 10.3389/fragi.2026.1838979

**Published:** 2026-07-22

**Authors:** Hao Mou, Chang-Jun Zhang

**Affiliations:** Beijing Key Laboratory of Ophthalmic Cell and Gene Therapy, Beijing Institute of Ophthalmology, Beijing Tongren Eye Center, Beijing Tongren Hospital, Capital Medical University, Beijing, China

**Keywords:** aging diseases, age-related macular degeneration, frailty, frailty components, nervous system diseases, UK Biobank

## Abstract

**Background:**

To explore the association between age-related macular degeneration (AMD) and frailty and the link between frailty and neurological aging diseases in AMD patients

**Methods:**

This study included 354,334 individuals aged ≥40 years without AMD at baseline from the UK Biobank. Frailty was assessed using the Fried phenotype. Cox proportional hazard models were used to analyze the association between frailty and AMD incidence. Logistic regression models were used to examine the link between frailty and nervous system-related diseases among AMD patients.

**Results:**

Frailty was associated with AMD incidence (hazard ratio [HR] 1.36 [95% confidence interval [95% CI] 1.29–1.44] for prefrailty; HR 2.12 [95% CI 1.86–2.42] for frailty). All five frailty components were associated with AMD: weight loss (HR 1.11 [95% CI 1.03–1.20]), exhaustion (HR 1.13 [95% CI 1.03–1.23]), low physical activity (HR 1.12 [95% CI 1.01–1.23]), low grip strength (HR 1.23 [95% CI 1.14–1.32]), and slow walking pace (HR 1.23 [95% CI 1.12–1.35]). Additionally, among AMD patients, frailty was linked to neurodegenerative diseases (odds ratio [OR] 3.09 [95% CI 1.58–6.07]), neurovascular diseases (OR 3.77 [95% CI 2.16–6.60]), and mental diseases (OR 2.09 [95% CI 1.12–3.93]).

**Conclusion:**

Frailty is a significant risk factor for AMD incidence, with all five components showing a positive association with AMD. Furthermore, frailty is linked to neural system-related diseases in AMD patients. Early detection and intervention for frailty may help prevent or delay AMD onset.

## Introduction

Age-related macular degeneration (AMD) is one of the major causes of visual impairment, predominantly affecting the older population (Guymer and Campbell, 2023). The number of AMD patients has been consistently increasing (Guymer and Campbell, 2023). In 2020, a study reported that AMD affected 196 million people worldwide and estimated that the number of patients would increase to 288 million by 2040 (Wong et al., 2014). The epidemiological investigation indicated that the incidence of AMD increased with population aging (Rudnicka et al., 2012). A meta-analysis calculating AMD prevalence in populations of European ancestry reported that the prevalence of late AMD was 1.4% at 70 years of age, 5.6% at 80 years of age, and 20% at 90 years of age (Rudnicka et al., 2012). The incidence of AMD was influenced by several risk factors, such as smoking (Seddon et al., 1996; Christen et al., 1996), physical activity (Mauschitz et al., 2022), lipids (Reynolds et al., 2010), and genetic predispositions (Ratnapriya et al., 2019; Fritsche et al., 2016). AMD is increasingly becoming a pressing public health issue. The disease not only impairs vision but also significantly impacts the quality of life, contributing to physical limitations and psychological distress. Given its high prevalence and substantial impact on well-being, AMD warrants further in-depth investigation.

Frailty was recognized as an age-related state with adverse health outcomes, and the degree of frailty was positively correlated with the severity of disease (Howlett et al., 2021; Thillainadesan et al., 2020). Although there were various classifications of frailty, the definition proposed by Fried and colleagues (Fried et al., 2001) was the most cited. Frailty contained five criteria: weight loss, exhaustion, physical activity, walking speed, and grip strength (Fried et al., 2001). The frailty status was recognized as individuals meeting three or more criteria, and people with one or two indicators were recognized as prefrailty status (Fried et al., 2001). Few studies reported the association between AMD and frailty directly. Several studies found that the association between dementia, one of the typical aging diseases, and frailty was intimate (Petermann-Rocha et al., 2020; Wallace et al., 2019). These results indicated that frailty was an essential indicator of aging (Wallace et al., 2019), and AMD was also recognized as an age-related disease (Rudnicka et al., 2012). Therefore, we wanted to investigate the association between frailty and AMD in middle-aged and older people. Moreover, based on previous studies, frailty was closely correlated with nervous system diseases. Herein, we sought to explore the association between frailty and nervous system diseases among AMD patients.

Here, we used data from the UK Biobank to explore the association between frailty and AMD. The UK Biobank recruited more than 500,000 participants from 2006 to 2010 across 22 assessment centers in England, Scotland, and Wales (Bycroft et al., 2018). All participants provided written informed consent and completed a series of physical assessments and questionnaires on topics such as lifestyle, sociodemographic characteristics, and medical history (Bycroft et al., 2018). Participants also provide biological samples for biochemical analysis, such as glucose, triglycerides, and cholesterol. Furthermore, among the included participants, we analyzed the participants who developed AMD during the following period and investigated the association between frailty and neurological diseases. The body mass index (BMI), age, and sex factors were considered as subgroup factors in analyzing the association between frailty and AMD.

## Methods

### Study design and participants

**FIGURE 1:**
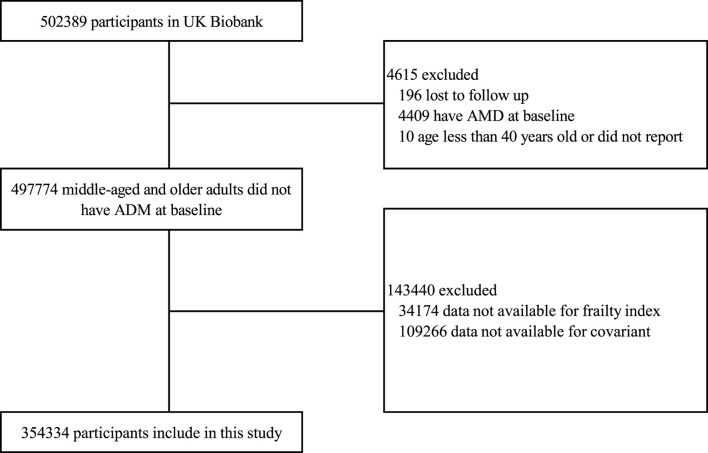
Flowchart of the study population. AMD, age-related macular degeneration.

In this study, all participants’ data were sourced from the UK Biobank database, and we excluded participants with AMD at baseline. The exclude data of baseline AMD patients were based on self-reported cases who received the AMD diagnosis reported by a touch screen questionnaire (field identification, 6,148) and a verbal interview (field identification, 20,001; code 1528) and the diagnostic criteria (International Classification of Diseases 10 [ICD-10; field identifications, 41202 and 41204; code H35.3] and International Classification of Diseases 9 [ICD-9; field identifications, 41203 and 41205; code 3625]). We also excluded participants who had withdrawn from the study or were younger than 40 years old due to our focus on middle-aged and older adults. We further excluded participants who did not have complete data for the frailty index or covariates. The remaining participants were included in the final analysis. The selection process is presented in Figure 1.

All participants provided written informed consent. The UK Biobank was approved by the North West Multi-centre Research Ethics Committee (reference 21/NW/0157). The study protocol of the UK Biobank was available online (https://biobank.ndph.ox.ac.uk/ukb/index.cgi).

### Frailty

The frailty was recognized as previously reported (Fried et al., 2001), which contained five criteria: weight loss, exhaustion, physical activity, walking speed, and grip strength. These data were collected from the self-reported and physical measurements of participants (Hanlon et al., 2018). Participants were classified as having frailty if they met three or more of five criteria, prefrailty if they met one or two criteria, and robust (nonfrail) if they met none of the criteria.

### Outcomes

The primary outcome was the incidence of AMD. AMD was defined by ICD-10 (field identifications, 41,202 and 41,204; code H35.3) and ICD-9 (field identifications, 41,203 and 41,205; code 3625) diagnoses for macular degeneration, as well as self-reported macular degeneration (field identification, 20,002; code 1528) (Z et al., 2022).

The secondary outcomes were neurological diseases diagnosed after AMD, including neurodegenerative diseases, neurovascular diseases, and mental diseases. We defined neurodegenerative diseases (Hou et al., 2019) (dementia, Parkinson’s disease, Alzheimer’s disease, Huntington’s disease, motor neuron disease, degenerative diseases of basal ganglia, multiple sclerosis, transient cerebral ischemic, myasthenia gravis, muscular dystrophy, spastic paraplegia, and other degenerative diseases of the nervous system), neurovascular diseases (subarachnoid hemorrhage, intracerebral hemorrhage, cerebral infarction, stroke, occlusion and stenosis of the precerebral arteries, occlusion and stenosis of the cerebral arteries, and other cerebrovascular diseases), and mental diseases (Momen et al., 2020) (mood, neurotic, stress-related, and somatoform disorders). These diseases were neural system-related diseases, and the field IDs are shown in Supplementary Material Table S1. Only neurological events with a diagnosis date subsequent to the index date of AMD were included.

### Covariates

We calculated age from the date of birth to the date of recruitment and subsequently classified participants into three age groups at 10-year intervals (Zhu et al., 2019): 40–50 years, 50–60 years, and older than 60 years. Ethnicity was self-reported, and we combined ethnicity groups into White and non-White. Education was classified into four categories: college (college or university); secondary education (advanced levels [A levels]/advanced subsidiary levels [AS levels] or equivalent, ordinary levels [O levels]/General Certificate of Secondary Education [GCSE] or equivalent, and Certificate of Secondary Education [CSE] or equivalent); professional level (National Vocational Qualification [NVQ] or Higher National Diploma [HND] or Higher National Certificate [HNC] or equivalent and other professional qualifications); and other (none of the above). The Townsend deprivation index was determined according to the census-based index of material deprivation (Townsend, 1987). The index contains four census variables: employment status, home ownership, car ownership, and household condition (Townsend, 1987). The higher index indicated the greater deprivation of this area. We categorized the index into three groups from low to high: lower (Townsend deprivation index from −6.26 to −3.14), middle (Townsend deprivation index from −3.14 to −0.589), and higher (Townsend deprivation index from −0.589 to 11). The income of participants was divided into five criteria from low to high (Said et al., 2018): less than £18,000 for an annual household, £18,000–30,999, £31,000–51,999, £52,000–100,000, and more than £100,000 annually. Smoking and alcohol intake status was self-reported and categorized. Smoking was classified into three categories: no (never smoke), previous (smoke previously), and yes (smoke currently). Alcohol consumption was divided into five categories by drinking frequency: never (never consumed alcohol), occasion (consumed alcohol on special occasions), 1–3 times per month, 1–4 times per week, and daily (consumed alcohol daily or almost daily). A healthy diet score was counted as previously reported (Said et al., 2018). The diet was categorized into 10 dietary components and a definite intake goal for each component. Overall, the diet score was categorized into ideal (adequate intake of >5 dietary components) or poor (inadequate intake of >5 dietary components). BMI was calculated (Frankel and Staeheli, 1992). In this study, BMI was classified into four levels, and the WHO criteria were applied (Obesity, 2000): thin (<18.5 kg/m^2^), normal (18.5–24.9 kg/m^2^), overweight (25–29.9 kg/m^2^), and obesity (≥30 kg/m^2^). Hours of sleep were self-reported and categorized as short (≤6 h), normal (6–9 h), and long sleep (≥9 h) as previously reported (Daghlas et al., 2019). The AMD polygenic risk score (AMD-PRS) was extracted from the UK Biobank (field identification, 26,204) and divided into four groups according to the quartile based on all participants. The first level was the least AMD-PRS (−4.32 to −0.586), the second was −0.586 to 0.113, the third was 0.113–0.822, and the fourth was 0.822–4.67. Diabetes, hypertension, and hyperlipidemia were defined by ICD-10 and ICD-9 and were self-reported, and the field IDs are shown in Supplementary Material Table S1. Only participants with complete data for all covariates were included in the study.

### Statistical analysis

Descriptive data were presented as the mean with standard deviation (SD) for continuous variables, which were normally distributed. And the data that were non-normally distributed were represented as the median and interquartile range (IQR). Categorical variables were represented as frequencies and percentages. R language software (version 4.3.1) was used for data analyses.

To analyze the baseline characteristics of the frailty category, we used the chi-square test to investigate the statistical difference of categorical variables. We compared the baseline characteristics of overall participants, robust, prefrailty, and frailty participants.

Association between frailty and AMD incidence was analyzed by the Cox proportional hazard models. The reference group was used as the nonfrailty participants. Results were represented as hazard ratios (HRs) with 95% confidence intervals (CIs). In addition, the association between AMD and the five components of frailty was investigated by the Cox proportional hazard models. Nonlinear associations between the number of individual components of frailty and AMD were tested by cubic splines based on the Cox proportional hazard models.

In order to obtain a more stable causal relationship, the sensitivity analysis was used, and the participants who had AMD within the 2 years of follow-up were excluded. In this sensitivity analysis, the Schoenfeld residuals were used to check the proportional hazard assumptions.

The neural system-related diseases were classified into three categories due to the few included participants: neurodegenerative diseases, neurovascular diseases, and mental diseases. We used the logistic regression model to investigate the association between frailty and neural system-related diseases among AMD patients. We analyzed the association between neural system-related disease and different statuses of frailty. Results were represented as odds ratios (ORs) with 95% CIs.

We ran three models for each outcome based on the previous study (Petermann-Rocha et al., 2020). Model 1 was minimally adjusted, which did not adjust any parameters. Model 2 included sociodemographic covariates, such as age, sex, deprivation, ethnicity, education, and income. Model 3 additionally included BMI, lifestyle factors (smoking, drinking, sleep, and diet), and disease status (AMD-PRS, hypertension, diabetes, and hyperlipidemia). Risk difference from model 1 was calculated as (HR_model 2_ − HR_model 1_)/(HR_model 1_ − 1) × 100%.

To investigate the association between frailty and AMD by subgroup, the models were run by adjusting sociodemographic covariates (age, sex, deprivation, ethnicity, education, and income); lifestyle factors (smoking, drinking, sleep, and diet); disease status (AMD-PRS, hypertension, diabetes, and hyperlipidemia); and BMI. The subgroup analyses by age–sex, age–weight, and weight–sex were represented in this study. In addition, the subgroup investigation of blood biochemical indicators was presented as HRs with 95% CIs. We did not adjust the corresponding diseases in the covariate for the subgroup analysis of the biochemistry item. For example, we did not adjust the covariates of diabetes when we analyzed the glucose.

## Results

The baseline characteristics of participants are presented in Supplementary Material Table S2. Among 354,334 participants, 215,763 were recognized as being robust, 128,268 participants were classified as having prefrailty, and 10,303 participants were identified as having frailty (Supplementary Material Table S3). Compared with robust people, frail participants were older, predominantly female, and non-White. Frail participants had less formal education and income with a higher Townsend deprivation index. As for the lifestyle, frail participants exhibited higher rates of smoking, reduced drinking, and lower-quality dietary patterns. Additionally, frail participants had higher BMI scores and were more likely to have longer or shorter sleep times. As for the AMD risk factors and chronic diseases, frail people had higher AMD-PRS scores and were more likely to suffer from chronic diseases, such as diabetes, hypertension, and hyperlipidemia (Supplementary Material Table S2). We collected the baseline characteristics stratified by AMD participants and represented in the appendix (Supplementary Material Table S3). Moreover, we presented the characteristics by individual frailty component (Supplementary Material Table S4).

The associations between the frailty status and AMD incidence are shown in Table 1. The HR was 1.36 (95% CI 1.29–1.44) for prefrailty and 2.12 (95% CI 1.86–2.42) for frailty in minimally adjusted model 1. The strength of these associations was attenuated for frailty if the model further adjusted sociodemographic covariates, such as age, sex, deprivation, ethnicity, education, and income (model 2: HR 1.25 [95% CI 1.18–1.32] for prefrailty and HR 1.65 [95% CI 1.44–1.89] for frailty). Additionally, the associations were more attenuated for prefrailty and frailty after adjusting for lifestyle factors, disease status, and BMI in model 3 (HR 1.18 [95% CI 1.11–1.25] for prefrailty and HR 1.41 [95% CI 1.23–1.62] for frailty, Table 1). Sensitivity analysis, which excluded participants who had AMD within the 2 years of follow-up, also indicated similar results (Table 2). Prefrailty and frailty were associated with AMD incidence. The HR was 1.37 (95% CI 1.29–1.45) for prefrailty and 2.09 (95% CI 1.83–2.40) for frailty in minimally adjusted model 1 (Table 2). The adjustment of confounders in the sensitivity analysis was the same as the investigation between frailty and AMD. The associations were attenuated in models 2 and 3 after adjusting for sociodemographic covariates and lifestyle factors, disease status, and BMI, respectively (model 2: HR 1.25 [95% CI 1.18–1.33] for prefrailty and HR 1.63 [95% CI 1.42–1.87] for frailty; model 3: HR 1.19 [95% CI 1.12–1.26] for prefrailty and HR 1.39 [95% CI 1.21–1.60] for frailty).

**TABLE 1:** Risk of AMD with frailty status.

Model	Prefrailty (*n* = 149,729)			Frailty (*n* = 12,876)		
	HR (95% CI)	p-value	Risk difference from model 1 %	HR (95% CI)	p-value	Risk difference from model 1, %
Model 1	1.36 (1.29,1.44)	<0.001	​	2.12 (1.86,2.42)	<0.001	​
Model 2	1.25 (1.18,1.32)	<0.001	−30.56%	1.65 (1.44,1.89)	<0.001	−41.96%
Model 3	1.18 (1.11,1.25)	<0.001	−50.00%	1.41 (1.23,1.62)	<0.001	−63.39%

The total number of participants was 354,334. Nonfrail participants were used as the reference group. Model 1 was minimally adjusted, which did not adjust any parameter. Model 2 included sociodemographic covariates: age, sex, deprivation, ethnicity, education, and income. Model 3 additionally included BMI, lifestyle factors (smoking, drinking, sleep, and diet), and disease status (AMD-PRS, hypertension, diabetes, and hyperlipidemia).

**TABLE 2:** Sensitivity analysis of AMD with frailty status.

Model	Prefrailty (*n* = 128,182)			Frailty (*n* = 10,288)			p for trend
	HR (95% CI)	p-value	Risk difference from model 1, %	HR (95% CI)	p-value	Risk difference from model 1, %	
Model 1	1.37 (1.29, 1.45)	<0.001	​	2.09 (1.83, 2.40)	<0.001	​	<0.001
Model 2	1.25 (1.18, 1.33)	<0.001	−32.43%	1.63 (1.42, 1.87)	<0.001	−42.20%	<0.001
Model 3	1.19 (1.12, 1.26)	<0.001	−48.65%	1.39 (1.21, 1.60)	<0.001	−64.22%	<0.001

The total number of participants was 354,111. Participants who experienced AMD within the 2 years of follow-up were excluded. Nonfrail participants were used as the reference group. Model 1 was minimally adjusted, which did not adjust any parameter. Model 2 included sociodemographic covariates: age, sex, deprivation, ethnicity, education, and income. Model 3 additionally included BMI, lifestyle factors (smoking, drinking, sleep, and diet), and disease status (AMD-PRS, hypertension, diabetes, and hyperlipidemia).

We investigated the association between the five components of frailty and AMD, and the results are represented in the appendix (Supplementary Material Table S5). The weight loss (HR 1.11 [95% CI 1.03–1.20]), tiredness (HR 1.13 [95% CI 1.03–1.23]), low physical activity level (HR 1.12 [95% CI 1.01–1.23]), low grip strength (HR 1.23 [95% CI 1.14–1.32]), and low gait speed (HR 1.23 [95% CI 1.12–1.35]) were all independently associated with the risk of AMD incidence (model 3; Supplementary Material Table S5). We found that the AMD incidence showed a dose-like association, although the evidence of a nonlinear association between frailty components and AMD incidence could not be found (Supplementary Material Figure S1).

We further investigated the association between frailty and nervous system-related diseases after participants developed AMD (Table 3). In this study, the nervous system-related diseases were categorized into three groups: neurodegenerative diseases, neurovascular diseases, and mental diseases. We found that the ORs of these diseases were increased markedly for participants with prefrailty and frailty, and frailty was associated with neurodegenerative diseases (frailty group, model 3: rate 5.71% OR 3.09 [95% CI 1.58–6.07]), neurovascular diseases (rate 9.39% OR 3.77 [95% CI 2.16–6.60]), and mental diseases (rate 6.12% OR 2.09 [95% CI 1.12–3.93]; Table 3). In addition, we investigated the association between frailty and the biochemical indicators of chronic diseases, including high blood pressure, glucose, triglycerides, hemoglobin concentration, and cholesterol (Supplementary Material Table S6). Although the association between frailty and with/without abnormal biochemical indicators could not be found, the risks of abnormal biochemical indicators were increased with prefrailty or frailty status (Supplementary Material Table S6).

**TABLE 3:** Odds ratios for neurodegenerative diseases, neurovascular diseases, and mental disorders in participants with frailty.

Frailty status	Total	Case	Rate	Model 1	Model 2	Model 3
Neurodegenerative diseases						
Robust	2,698	45	1.67%	​	​	​
Prefrailty	2,120	69	3.25%	1.98 (1.36,2.90)	1.91 (1.30,2.81)	1.83 (1.23,2.71)
Frailty	245	14	5.71%	3.57 (1.93,6.61)	3.30 (1.74,6.28)	3.09 (1.58,6.07)
Neurovascular diseases						
Robust	2,698	62	2.30%	​	​	​
Prefrailty	2,120	86	4.06%	1.80 (1.29,2.50)	1.83 (1.31,2.57)	1.67 (1.18,2.36)
Frailty	245	23	9.39%	4.40 (2.68, 7.25)	4.78 (2.83, 8.07)	3.77 (2.16, 6.60)
Mental disorder						
Robust	2,698	66	2.45%	​	​	​
Prefrailty	2,120	79	3.73%	1.54 (1.11, 2.15)	1.50 (1.07, 2.10)	1.40 (0.99, 1.98)
Frailty	245	15	6.12%	2.60 (1.46, 4.63)	2.45 (1.34, 4.46)	2.09 (1.12, 3.93)

The association between three categories of age-related diseases and the prefrailty and frailty statuses. This table includes participants with AMD and examines the association between prefrailty and frailty status and the risk of secondary neurological diseases. Model 1 was minimally adjusted, which did not adjust any parameter. Model 2 included sociodemographic covariates: age, sex, deprivation, ethnicity, education, and income. Model 3 additionally included BMI, lifestyle factors (smoking, drinking, sleep, and diet), and disease status (AMD-PRS, hypertension, diabetes, and hyperlipidemia). Data were odds ratios (95% CI) in different models.

**FIGURE 2:**
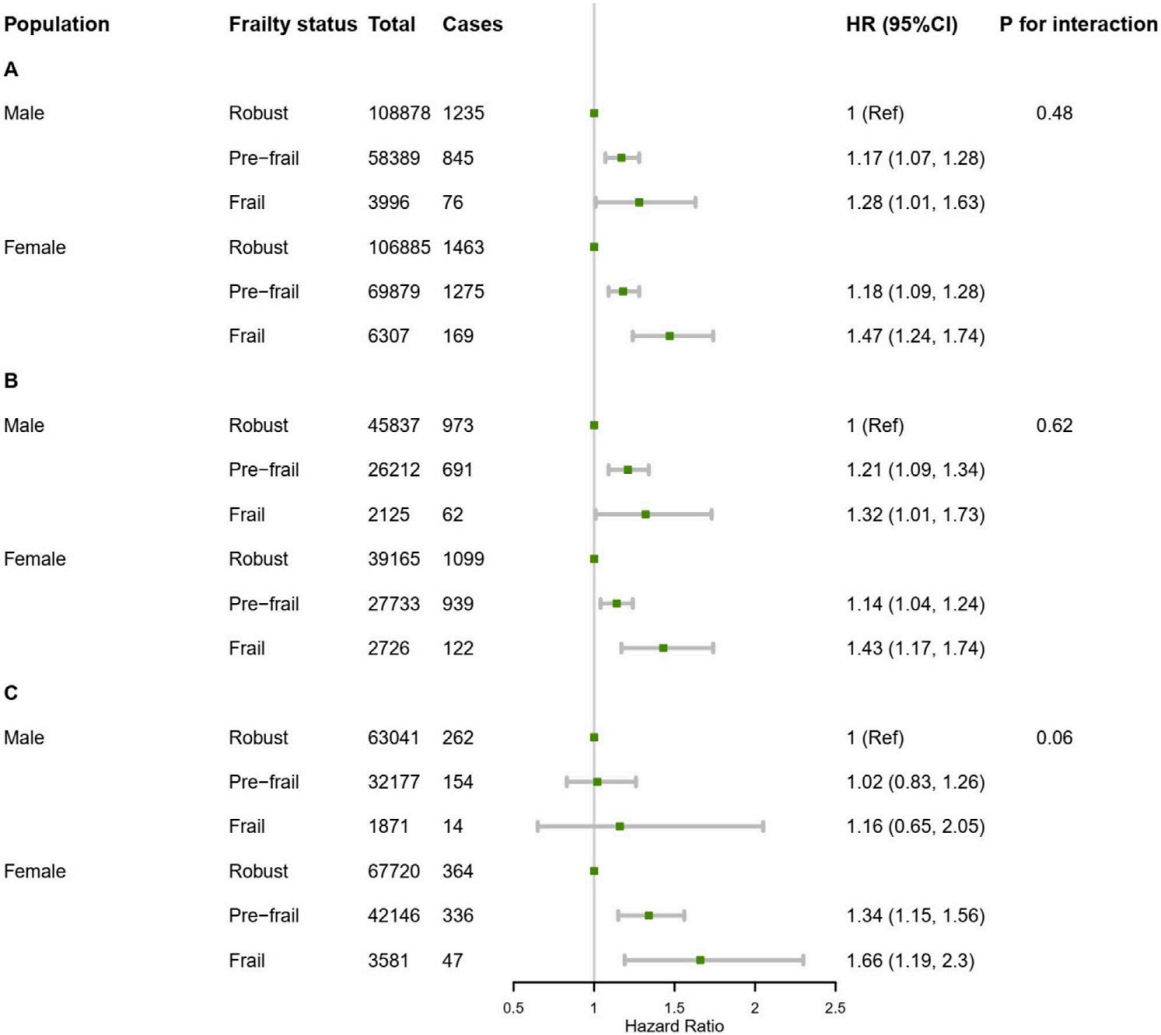
Association of baseline frailty status with AMD by sex. Notes: nonfrail people were used as the reference group for each subgroup. Analyses were adjusted by age, sex, education, ethnicity, deprivation, income, smoking, drinking, sleep, diet, AMD-PRS, hypertension, diabetes, and hyperlipidemia. HR = hazard ratio. **(A)** Overall population. **(B)** Old adults. **(C)** Middle-aged adults.

**FIGURE 3:**
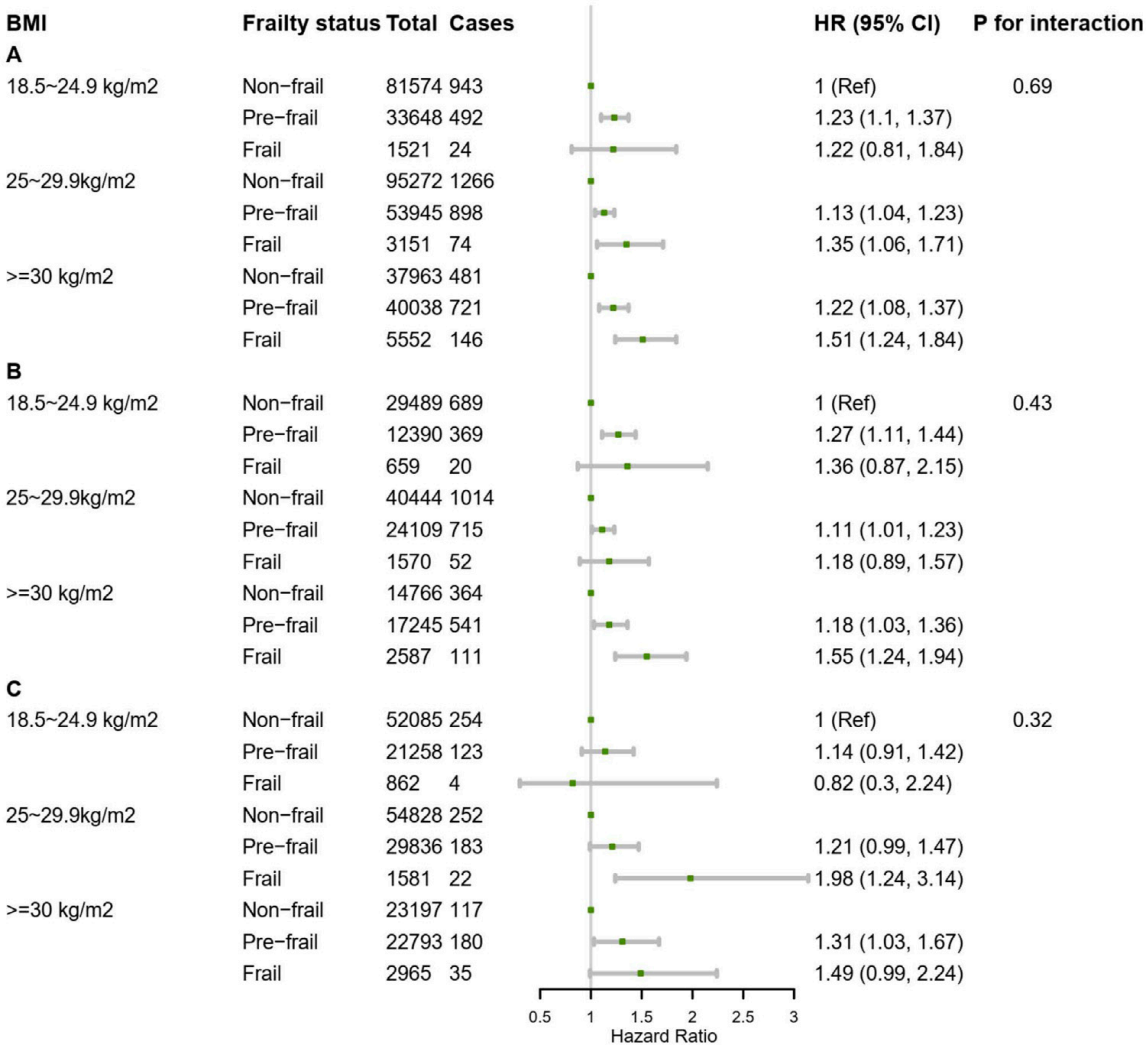
Association of baseline frailty status with AMD by BMI. Notes: nonfrail people were used as the reference group for each subgroup. Analyses were adjusted by age, education, ethnicity, deprivation, income, BMI, smoking, drinking, sleep, diet, AMD-PRS, hypertension, diabetes, and hyperlipidemia. HR = hazard ratio. **(A)** Overall population. **(B)** Old adults. **(C)** Middle-aged adults.

We investigated the association between frailty and AMD by subgroup analyses, and groups of age–sex, age–weight, and weight–sex were represented in this study (Figures 2, 3; Supplementary Material Figure S2). We noticed that the HR of the female population was higher than that of males in the association of baseline frailty status with incident AMD, and the gender difference was more pronounced in the middle-aged population (Figure 2). As for the age–weight subgroup analysis, people with obesity under frailty status had a stronger association with AMD, and the obesity factor mainly affected the older adults (Figure 3). The subgroup analysis between gender and BMI was further investigated, and the HR of the female population was also higher than that of the male population (Supplementary Material Figure S2).

## Discussion

In this study, we identified that individuals with prefrailty and frailty had an increased risk of AMD incidence compared with nonfrail individuals. In addition, AMD patients with prefrailty or frailty were more likely to suffer from diseases of the nervous system. Through subgroup analysis, the association between frailty and AMD indicated a tendency in females or in individuals with obesity. This study clarified the association between frailty and the incidence of AMD. These findings might help prevent or delay the onset of AMD and other aging-related diseases.

Based on the large cohort of the UK Biobank, our results provided a robust conclusion on the association between frailty and the incidence of AMD. However, few studies reported the association between frailty and the incidence of AMD systematically. A cross-sectional study reported the association between frailty and several ophthalmic conditions, including glaucoma suspect, intraocular pressure, diabetic retinopathy, AMD, and cataract (Ghanbarnia et al., 2023). Although in the fully adjusted binary logistic regression model, the association between physical frailty and ophthalmic conditions could not be found, the odds ratio of AMD and physical frailty was significant (p = 0.009) (Ghanbarnia et al., 2023). In 2005, the Beaver Dam Eye Study investigated the association between frailty and AMD in 2,962 participants. The association between handgrip strength and AMD was found in males by analyzing the odds ratio, while the association between frailty and late AMD was not found in female participants (Klein et al., 2005). In our study, we found that all components of frailty, not just the handgrip strength, were associated with the occurrence of AMD. There might be two factors that lead to further results in our study: one was that more participants were included in our study, and the other factor was that the prospective cohort study was used to investigate the incidence of AMD among older people. In addition, we investigated the association between the five components of frailty and AMD. All components were associated with AMD in model 3, which was maximally adjusted (Supplementary Material Table S5). Our data suggested the role of frailty in AMD development. Previous studies reported that gait speed and grip strength were associated with age-related cataract (Klein et al., 2006); hand grip strength was associated with visual impairment (Klein et al., 2005; Shang et al., 2022). Moreover, the high level of physical activity protected against the development of early AMD (Mauschitz et al., 2022). In our study, the scope of risk factors had expanded, which indicated that all components of frailty were associated with AMD. All results suggested that each component of frailty was associated with an increased risk of AMD, and as an aging indicator, its changes might have a remarkable effect on aging.

We investigated the association between frailty and neurological diseases among participants after AMD onset (Table 3). Different from other studies focusing solely on the association between frailty and AMD (Zhu et al., 2025; Peng et al., 2026), our data further indicated that after AMD developed, frailty was more strongly associated with neurodegenerative, neurovascular, and mental diseases (Table 3). As previously reported, frailty was associated with age-related diseases, such as dementia and Alzheimer’s disease (Petermann-Rocha et al., 2020; Wallace et al., 2019; Panza et al., 2019; Rogers et al., 2017). Our results were consistent with previous reports, as AMD was also recognized as an age-related disease (Petermann-Rocha et al., 2020; Wallace et al., 2019). Numerous studies reported that during frailty and aging, metabolic disorder and inflammatory responses were the causes of neurological diseases (Blacher et al., 2022; Minhas et al., 2021; Ma and Chan, 2020; Pan and Ma, 2024; Hansman et al., 2025). Specifically, reduced grip strength and slower walking speed reflected systemic chronic inflammation and impaired microcirculation, which were evaluated as toxic factors in blood and further propagate neuroinflammatory cascades underlying neurological disorders (Westbrook et al., 2020; Abadir et al., 2017). Declined physical activity exacerbated systemic inflammation responses and oxidative stress, accelerating retinal degenerative changes and aggravating central nervous system diseases (Szilagyi et al., 2024; Chu-Tan et al., 2022). Weight loss and persistent fatigue mirrored mitochondrial dysfunction and disturbed lipid metabolism, impairing the metabolic homeostasis required for both retinal and neural cell survival (Hansman et al., 2025; Kocherlakota et al., 2023; Kaarniranta et al., 2020; Li et al., 2022). Notably, reduced microglial homeostasis could lead to cellular abnormal metabolism and activation of inflammatory response in retinal tissues (Blacher et al., 2022; Minhas et al., 2021). By using single-cell sequencing, similar abnormal microglia activation mediated by interleukin-1β was observed both in humans and mice during AMD progression (Kuchroo et al., 2023). These studies indicated the role of microglia in linking frailty, neurological diseases, and AMD. In our study, we illustrated that frailty increased the risk of neurological diseases after AMD onset, and exploring the mechanical role of frailty components might be a direction for further research.

Multiple factors interacted with AMD, such as diabetes, hyperlipidemia, smoking, obesity, and genetic factors (Guymer and Campbell, 2023; Seddon et al., 1996; Christen et al., 1996; Mauschitz et al., 2022; Reynolds et al., 2010; Ting et al., 2017; Clemons et al., 2005). By subgroup analysis, we investigated the association between biochemical indicators and AMD (Supplementary Material Table S6). High blood pressure, glucose, triglycerides, hemoglobin concentration, and total cholesterol were the core of hypertension, diabetes, hyperlipidemia, anemia, and hyperuricemia, respectively. Unfortunately, the significant association could not be found (Supplementary Material Table S6). Our results suggested that core biochemical indicators might not affect the association between frailty and AMD. It might be due to the strong feedback networks of the biochemical factors, which weaken the interaction effect. We also investigated other factors in the study and found that females had a higher HR in the association of frailty and AMD (Figure 2; Supplementary Material Figure S2). Besides, we found that AMD or frailty status appeared to be more pronounced in female participants (Supplementary Material Table S2). These results were consistent with reported studies (Petermann-Rocha et al., 2020; Hanlon et al., 2018; Clemons et al., 2005; Jonasson et al., 2014). By analyzing the expression changes between males and females in four brain regions, the earlier age-related expression changes were observed in the female superior frontal gyrus, which is related to stress and the higher prevalence of Alzheimer’s disease (Yuan et al., 2012). Moreover, the basic research on mice indicated that the inflammatory markers were increased in the brain of aging mice (Hahn et al., 2023). These results may partly explain the tendency for females to exhibit greater vulnerability to biological aging and age-related diseases.

Our study had several strengths. First, we investigated a large, prospective cohort with plenty of research resources based on the UK Biobank. As a result, we could adjust a wide range of sociodemographic and lifestyle factors when performing analysis. Second, to our knowledge, this was the first study to examine the association between frailty and AMD systematically. Specifically, we investigated the association between frailty and age-related neural system diseases after AMD developed. Therefore, our results filled the gap between neurology and ophthalmology in aging research. Third, these findings might be helpful for the AMD intervention measures, AMD treatment, scientific basis, and reference for decision-makers in the future.

We also acknowledged that our study had some limitations. First, information on the classification and the degree of AMD was unavailable in the UK Biobank. Specifically, the classification of AMD, such as early AMD and late AMD or wet AMD and dry AMD, was unable to be obtained. We could not focus on the disease stage of AMD or investigate the association between different stages of AMD and frailty further. Second, we were unable to identify participants with AMD complications, such as choroidal neovascular, serous pigment epithelial detachment, and geographic atrophy (Guymer and Campbell, 2023). The database of the ICD-9, ICD-10, and self-reported cases in the UK Biobank could not provide the detailed complication information of AMD. Of note, AMD case ascertainment in this study relied on ICD-9/ICD-10 diagnostic codes and participant self-reports, without access to standardized ophthalmic clinical assessment or objective imaging evidence such as OCT and fundus photography. This approach inevitably led to potential misclassification bias: asymptomatic early AMD cases were prone to under-ascertainment in administrative coding and self-reported records, whereas the lack of imaging phenotyping prevents accurate identification of AMD pathological features. Third, we could not eliminate all confounding factors, as it was a large-scale retrospective cohort study, such as genetic factors and unmeasured lifestyle variables. Therefore, we considered confounding factors as carefully as possible and adjusted for confounding factors by using different models. Fourth, frailty components adopted in this study, such as grip strength and walking speed, also act as the surrogate markers of central nervous system integrity (Fried et al., 2001; Gao et al., 2026). Therefore, the observed associations between frailty and AMD may partially reflect shared underlying aging processes. Besides, frailty is a dynamic state and changes over time, which could influence the association between frailty and AMD (Hoogendijk et al., 2019). However, repeated frailty measurements were unavailable for sensitivity analyses due to the limited effective sample size.

In conclusion, frailty (both the prefrail and frail statuses) was associated with an increased risk of AMD incidence, and all five components were associated with AMD. Furthermore, age-related neural system diseases were associated with frailty after AMD developed. As one of the markers of aging, frailty demonstrated its important role in AMD development. Our findings might be helpful for aging research and provide possible methods for AMD intervention and treatment.

## Data Availability

The original contributions presented in the study are included in the article/Supplementary Material; further inquiries can be directed to the corresponding author.
