# Supplementary material for: Association between frailty and incidence of AMD among middle-aged and older people: evidence from the UK Biobank
Source: Front Aging. 2026 Jul 22;7:1838979. doi: 10.3389/fragi.2026.1838979 (PMC13438126; doi:10.3389/fragi.2026.1838979)
Supplement: Supplementary file 1 [file Table1.docx]

**Association between frailty and age-related macular degeneration in the UK Biobank**

**Supplementary materials**

**Supplementary Table 1. ICD codes and Field ID of diseases in the UK Biobank.**

**Supplementary Table 2. Baseline characteristics according to frailty category**

**Supplementary Table 3. Baseline characteristics of incidence AMD by baseline frailty status in the UK Biobank cohort.**

**Supplementary Table 4. Sociodemographic characteristics of the study population by the individual components of frailty at incidence AMD.**

**Supplementary Table 5. Individual components of frailty and their association with AMD incidence.**

**Supplementary Table 6. Association of baseline frailty status with AMD by health status.**

**Supplementary Figure 1. Non-linear associations between number of individual components of the frailty phenotype and AMD incidence**.

**Supplementary Figure 2. Association of baseline frailty status with AMD by sex.**

**Supplementary Table 1.** **ICD codes and Field ID of diseases in the UK Biobank**

| **Disease** | **ICD-10** | **ICD-9** | **Data field ID in UKB** |
| --- | --- | --- | --- |
| **Neurodegenerative diseases** |  | - |  |
| Dementia | F00-F03 | 290 | 1263 |
| Parkinson's disease | G20-G22 | 332 | 1262 |
| Alzheimer's disease | G30 | 3310 |  |
| Huntington's disease | G10 | 3334 |  |
| Motor neuron disease | G12 | 3352 | 1259 |
| Degenerative diseases of basal ganglia | G23 | 3330 |  |
| Other degenerative diseases of nervous system | G31 | 3311-3319 | 1258,1397 |
| Multiple sclerosis | G35 | 3409 | 1261 |
| Transient cerebral ischemic | G45 | 4359 |  |
| Myasthenia gravis | G70 | 3580,35809 | 1260 |
| Muscular dystrophy | G71 | 3351,3599 |  |
| Spastic paraplegia | G82 | 3341 |  |
| **Neurovascular diseases** |  |  |  |
| Cerebrovascular disease | I60-I69 | 430-438 | 1081,1082,1083,1086,1425,1491,1583 |
| **Mental diseases** |  |  |  |
| Schizophrenia, schizotypal and delusional disorders | F20-F29 | 295 |  |
| Mood (affective) disorders | F30-F39 | 296,311 | 1286,1290,1291 |
| Neurotic, stress-related and somatoform disorders | F40-F48 | 300 | 1287,1288 |
| Diabetes | E10-E14, G632, G590, H360, H280 | 250 | 1220,1222,1223,1276,1468,1607 |
| Hypertension | I10-I15 | 401-405 | 1065,1072 |
| Hyperlipidemia | E78 | 2720 | 1473 |

Note: The codes in this table were the highest level for this disease. All sub codes under this category were included in the codes in this table. Data field ID in the UK Biobank was from the self-report of participants, and the parent ID was 20002. ICD: international classification disease.

**Supplementary Table 2.** **Baseline characteristics according to frailty category**

|  | Overall | Robust | Pre-frailty | Frailty | P value |
| --- | --- | --- | --- | --- | --- |
| Total | 354334 | 215763 | 128268 | 10303 |  |
| Age |  |  |  |  |  |
| Age (years) | 56.05 (8.07) | 55.82 (8.05) | 56.31 (8.12) | 57.69 (7.61) | <0.001 |
| 40-50 years | 99321 (28.0) | 62623 (29.0) | 34631 (27.0) | 2067 (20.1) | <0.001 |
| 50-60 years | 128281 (36.2) | 78529 (36.4) | 45815 (35.7) | 3937 (38.2) |  |
| >60 years | 126732 (35.8) | 74611 (34.6) | 47822 (37.3) | 4299 (41.7) |  |
| Sex |  |  |  |  |  |
| Male | 171263 (48.3) | 108878 (50.5) | 58389 (45.5) | 3996 (38.8) | <0.001 |
| Female | 183071 (51.7) | 106885 (49.5) | 69879 (54.5) | 6307 (61.2) |  |
| Ethnicity |  |  |  |  |  |
| White | 325511 (91.9) | 199775 (92.6) | 116575 (90.9) | 9161 (88.9) | <0.001 |
| Other | 28823 (8.1) | 15988 (7.4) | 11693 (9.1) | 1142 (11.1) |  |
| Education |  |  |  |  |  |
| College | 127857 (36.1) | 85524 (39.6) | 40451 (31.5) | 1882 (18.3) | <0.001 |
| Secondary education | 137081 (38.7) | 82503 (38.2) | 50840 (39.6) | 3738 (36.3) |  |
| Professional level | 41107 (11.6) | 24069 (11.2) | 15712 (12.2) | 1326 (12.9) |  |
| Other | 48289 (13.6) | 23667 (11.0) | 21265 (16.6) | 3357 (32.6) |  |
| Deprivation |  |  |  |  |  |
| Lower | 118117 (33.3) | 78193 (36.2) | 38050 (29.7) | 1874 (18.2) | <0.001 |
| Middle | 118108 (33.3) | 73786 (34.2) | 41716 (32.5) | 2606 (25.3) |  |
| Higher | 118109 (33.3) | 63784 (29.6) | 48502 (37.8) | 5823 (56.5) |  |
| Income |  |  |  |  |  |
| ≤18000 | 73653 (20.8) | 35336 (16.4) | 32926 (25.7) | 5391 (52.3) | <0.001 |
| 18000-30999 | 89337 (25.2) | 52564 (24.4) | 34248 (26.7) | 2525 (24.5) |  |
| 31000-51999 | 94920 (26.8) | 60803 (28.2) | 32563 (25.4) | 1554 (15.1) |  |
| 52000-100000 | 76028 (21.5) | 52249 (24.2) | 23059 (18.0) | 720 (7.0) |  |
| ≥100000 | 20396 (5.8) | 14811 (6.9) | 5472 (4.3) | 113 (1.1) |  |
| Smoking |  |  |  |  |  |
| No | 193646 (54.7) | 121902 (56.5) | 67181 (52.4) | 4563 (44.3) | <0.001 |
| Previous | 124239 (35.1) | 74799 (34.7) | 45734 (35.7) | 3706 (36.0) |  |
| Yes | 36449 (10.3) | 19062 (8.8) | 15353 (12.0) | 2034 (19.7) |  |
| Drinking |  |  |  |  |  |
| Never | 22910 (6.5) | 10307 (4.8) | 10687 (8.3) | 1916 (18.6) | <0.001 |
| Occasion | 35733 (10.1) | 17115 (7.9) | 16233 (12.7) | 2385 (23.1) |  |
| 1-3 times per month | 38938 (11.0) | 21724 (10.1) | 15826 (12.3) | 1388 (13.5) |  |
| 1-4 times per week | 179175 (50.6) | 114608 (53.1) | 61150 (47.7) | 3417 (33.2) |  |
| Daily | 77578 (21.9) | 52009 (24.1) | 24372 (19.0) | 1197 (11.6) |  |
| Diet |  |  |  |  |  |
| Healthy diet score | 2.97 (1.39) | 2.98 (1.39) | 2.95 (1.40) | 2.80 (1.39) | <0.001 |
| Ideal | 47947 (13.5) | 29474 (13.7) | 17287 (13.5) | 1186 (11.5) | <0.001 |
| Poor | 306387 (86.5) | 186289 (86.3) | 110981 (86.5) | 9117 (88.5) |  |
| BMI |  |  |  |  |  |
| BMI | 27.34 (4.70) | 26.58 (4.11) | 28.29 (5.08) | 31.42 (6.75) | <0.001 |
| <18.5 | 1670 (0.5) | 954 (0.4) | 637 (0.5) | 79 (0.8) | <0.001 |
| 18.5-24.9 | 116743 (32.9) | 81574 (37.8) | 33648 (26.2) | 1521 (14.8) |  |
| 25-29.9 | 152368 (43.0) | 95272 (44.2) | 53945 (42.1) | 3151 (30.6) |  |
| ≥30 | 83553 (23.6) | 37963 (17.6) | 40038 (31.2) | 5552 (53.9) |  |
| Sleep |  |  |  |  |  |
| ≤6 hours | 83814 (23.7) | 44926 (20.8) | 34954 (27.3) | 3934 (38.2) | <0.001 |
| 6-9 hours | 245204 (69.2) | 158013 (73.2) | 82599 (64.4) | 4592 (44.6) |  |
| ≥9 hours | 25316 (7.1) | 12824 (5.9) | 10715 (8.4) | 1777 (17.2) |  |
| AMD |  |  |  |  |  |
| AMD-PRS | 0.13 (1.03) | 0.12 (1.03) | 0.13 (1.03) | 0.15 (1.04) | <0.001 |
| First (lowest) | 88584 (25.0) | 54256 (25.1) | 31906 (24.9) | 2422 (23.5) | <0.001 |
| Second | 88583 (25.0) | 54059 (25.1) | 31962 (24.9) | 2562 (24.9) |  |
| Third | 88583 (25.0) | 53919 (25.0) | 32009 (25.0) | 2655 (25.8) |  |
| Fourth (highest) | 88584 (25.0) | 53529 (24.8) | 32391 (25.3) | 2664 (25.9) |  |
| Disease |  |  |  |  |  |
| Diabetes | 17491 (4.9) | 6165 (2.9) | 9354 (7.3) | 1972 (19.1) | <0.001 |
| Hypertension | 97822 (27.6) | 50251 (23.3) | 42235 (32.9) | 5336 (51.8) | <0.001 |
| Hyperlipidemia | 51241 (14.5) | 26019 (12.1) | 22157 (17.3) | 3065 (29.7) | <0.001 |

Notes: The healthy diet score, BMI and AMD-PRS were represented as median (IQR), or mean (SD). Other data were n (%). Percentages might not add to 100% due to rounding. BMI=body-mass index. AMD=age-related macular degeneration. PRS=polygenic risk score.

**Supplementary Table 3. Baseline characteristics of incidence AMD by baseline frailty status in the UK Biobank cohort**

|  | AMD participants |
| --- | --- |
| Total | 5063 |
| Age |  |
| Age (years) | 62.61 (5.45) |
| 40-50 years | 203 (4.0) |
| 50-60 years | 1246 (24.6) |
| >60 years | 3614 (71.4) |
| Sex |  |
| Male | 2156 (42.6) |
| Female | 2907 (57.4) |
| Ethnicity |  |
| White | 4713 (93.1) |
| Other | 350 (6.9) |
| Education |  |
| College | 1455 (28.7) |
| Secondary education | 1752 (34.6) |
| Professional level | 709 (14.0) |
| Other | 1147 (22.7) |
| Deprivation |  |
| Lower | 1643 (32.5) |
| Middle | 1754 (34.6) |
| Higher | 1666 (32.9) |
| Income |  |
| ≤18000 | 1713 (33.8) |
| 18000-30999 | 1537 (30.4) |
| 31000-51999 | 1081 (21.4) |
| 52000-100000 | 629 (12.4) |
| ≥100000 | 103 (2.0) |
| Smoking |  |
| No | 2477 (48.9) |
| Previous | 2153 (42.5) |
| Yes | 433 (8.6) |
| Drinking |  |
| Never | 451 (8.9) |
| Occasion | 619 (12.2) |
| 1-3 times per month | 490 (9.7) |
| 1-4 times per week | 2292 (45.3) |
| Daily | 1211 (23.9) |
| Diet |  |
| Healthy diet score | 3.17 (1.40) |
| Ideal | 878 (17.3) |
| Poor | 4185 (82.7) |
| BMI |  |
| BMI | 27.80 (4.72) |
| <18.5 | 18 (0.4) |
| 18.5-24.9 | 1459 (28.8) |
| 25-29.9 | 2238 (44.2) |
| ≥30 | 1348 (26.6) |
| Sleep |  |
| ≤6 hours | 1191 (23.5) |
| 6-9 hours | 3387 (66.9) |
| ≥9 hours | 485 (9.6) |
| AMD |  |
| AMD-PRS | 0.47 (1.12) |
| Lower | 899 (17.8) |
| Middle | 1021 (20.2) |
| Middle-higher | 1252 (24.7) |
| Higher | 1891 (37.3) |
| Disease |  |
| Diabetes | 503 (9.9) |
| Hypertension | 2042 (40.3) |
| Hyperlipidemia | 1212 (23.9) |

Notes: The healthy diet score, BMI and AMD-PRS were represented as median (IQR), or mean (SD). Other data were n (%). BMI=body-mass index. AMD=age-related macular degeneration. PRS=polygenic risk score.

**Supplementary Table 4. Sociodemographic characteristics of the study population by the individual components of frailty at incidence AMD**

|  | Grip | Physical activity | Weight change | Walk pace | Tiredness |
| --- | --- | --- | --- | --- | --- |
| Total | 44830 | 27863 | 52490 | 23865 | 40676 |
| Age |  |  |  |  |  |
| Age (years) | 59.48 (7.21) | 55.75 (7.95) | 55.68 (8.13) | 58.71 (7.47) | 54.23 (7.95) |
| 40-50 years | 6182 (13.8) | 7950 (28.5) | 15479 (29.5) | 3953 (16.6) | 14418 (35.4) |
| 50-60 years | 14762 (32.9) | 10734 (38.5) | 19070 (36.3) | 8385 (35.1) | 15821 (38.9) |
| >60 years | 23886 (53.3) | 9179 (32.9) | 17941 (34.2) | 11527 (48.3) | 10437 (25.7) |
| Sex |  |  |  |  |  |
| Male | 18570 (41.4) | 11973 (43.0) | 24880 (47.4) | 11058 (46.3) | 16543 (40.7) |
| Female | 26260 (58.6) | 15890 (57.0) | 27610 (52.6) | 12807 (53.7) | 24133 (59.3) |
| Ethnicity |  |  |  |  |  |
| White | 40597 (90.6) | 24838 (89.1) | 48196 (91.8) | 21366 (89.5) | 36370 (89.4) |
| Other | 4233 (9.4) | 3025 (10.9) | 4294 (8.2) | 2499 (10.5) | 4306 (10.6) |
| Education |  |  |  |  |  |
| College | 12213 (27.2) | 6192 (22.2) | 18180 (34.6) | 5349 (22.4) | 11783 (29.0) |
| Secondary education | 16802 (37.5) | 11170 (40.1) | 20947 (39.9) | 8492 (35.6) | 16478 (40.5) |
| Professional level | 5610 (12.5) | 3558 (12.8) | 6238 (11.9) | 3091 (13.0) | 5041 (12.4) |
| Other | 10205 (22.8) | 6943 (24.9) | 7125 (13.6) | 6933 (29.1) | 7374 (18.1) |
| Deprivation |  |  |  |  |  |
| Lower | 12405 (27.7) | 5976 (21.4) | 16532 (31.5) | 5496 (23.0) | 10488 (25.8) |
| Middle | 14263 (31.8) | 7771 (27.9) | 17335 (33.0) | 6906 (28.9) | 12397 (30.5) |
| Higher | 18162 (40.5) | 14116 (50.7) | 18623 (35.5) | 11463 (48.0) | 17791 (43.7) |
| Income |  |  |  |  |  |
| ≤18000 | 15600 (34.8) | 9604 (34.5) | 11905 (22.7) | 10899 (45.7) | 12534 (30.8) |
| 18000-30999 | 12829 (28.6) | 7429 (26.7) | 13329 (25.4) | 6140 (25.7) | 10313 (25.4) |
| 31000-51999 | 9550 (21.3) | 6436 (23.1) | 13616 (25.9) | 4239 (17.8) | 9809 (24.1) |
| 52000-100000 | 5600 (12.5) | 3710 (13.3) | 10767 (20.5) | 2155 (9.0) | 6732 (16.6) |
| ≥100000 | 1251 (2.8) | 684 (2.5) | 2873 (5.5) | 432 (1.8) | 1288 (3.2) |
| Smoking |  |  |  |  |  |
| No | 23955 (53.4) | 12915 (46.4) | 27471 (52.3) | 10260 (43.0) | 21207 (52.1) |
| Previous | 16580 (37.0) | 9341 (33.5) | 19018 (36.2) | 9523 (39.9) | 13234 (32.5) |
| Yes | 4295 (9.6) | 5607 (20.1) | 6001 (11.4) | 4082 (17.1) | 6235 (15.3) |
| Drinking |  |  |  |  |  |
| Never | 4958 (11.1) | 3407 (12.2) | 4039 (7.7) | 3479 (14.6) | 4486 (11.0) |
| Occasion | 6638 (14.8) | 4834 (17.3) | 6504 (12.4) | 4376 (18.3) | 6415 (15.8) |
| 1-3 times per month | 5274 (11.8) | 3676 (13.2) | 6604 (12.6) | 3006 (12.6) | 5493 (13.5) |
| 1-4 times per week | 19892 (44.4) | 11174 (40.1) | 26069 (49.7) | 9126 (38.2) | 17577 (43.2) |
| Daily | 8068 (18.0) | 4772 (17.1) | 9274 (17.7) | 3878 (16.2) | 6705 (16.5) |
| Diet |  |  |  |  |  |
| Healthy diet score | 2.98 (1.39) | 2.58 (1.35) | 3.17 (1.38) | 2.78 (1.39) | 2.80 (1.40) |
| Ideal | 6256 (14.0) | 2337 (8.4) | 8728 (16.6) | 2682 (11.2) | 4661 (11.5) |
| Poor | 38574 (86.0) | 25526 (91.6) | 43762 (83.4) | 21183 (88.8) | 36015 (88.5) |
| BMI |  |  |  |  |  |
| BMI | 29.36 (5.19) | 29.51 (6.03) | 27.81 (4.95) | 31.36 (6.57) | 28.65 (5.65) |
| <18.5 | 211 (0.5) | 148 (0.5) | 357 (0.7) | 103 (0.4) | 268 (0.7) |
| 18.5-24.9 | 8315 (18.5) | 6236 (22.4) | 15334 (29.2) | 3460 (14.5) | 10799 (26.5) |
| 25-29.9 | 18307 (40.8) | 10224 (36.7) | 22600 (43.1) | 7700 (32.3) | 15762 (38.8) |
| ≥30 | 17997 (40.1) | 11255 (40.4) | 14199 (27.1) | 12602 (52.8) | 13847 (34.0) |
| Sleep |  |  |  |  |  |
| ≤6 hours | 12218 (27.3) | 9110 (32.7) | 13537 (25.8) | 7503 (31.4) | 14497 (35.6) |
| 6-9 hours | 28106 (62.7) | 15837 (56.8) | 35111 (66.9) | 12627 (52.9) | 21499 (52.9) |
| ≥9 hours | 4506 (10.1) | 2916 (10.5) | 3842 (7.3) | 3735 (15.7) | 4680 (11.5) |
| AMD |  |  |  |  |  |
| AMD-PRS | 0.14 (1.03) | 0.15 (1.03) | 0.13 (1.03) | 0.14 (1.03) | 0.14 (1.03) |
| Lower | 10882 (24.3) | 6808 (24.4) | 13087 (24.9) | 5878 (24.6) | 10060 (24.7) |
| Middle | 11255 (25.1) | 6909 (24.8) | 13132 (25.0) | 5874 (24.6) | 10091 (24.8) |
| Middle-higher | 11246 (25.1) | 7009 (25.2) | 13132 (25.0) | 6029 (25.3) | 10325 (25.4) |
| Higher | 11447 (25.5) | 7137 (25.6) | 13139 (25.0) | 6084 (25.5) | 10200 (25.1) |
| Disease |  |  |  |  |  |
| Diabetes | 4559 (10.2) | 2816 (10.1) | 4781 (9.1) | 3628 (15.2) | 3380 (8.3) |
| Hypertension | 17655 (39.4) | 10262 (36.8) | 17662 (33.6) | 11964 (50.1) | 13456 (33.1) |
| Hyperlipidemia | 9979 (22.3) | 5567 (20.0) | 8996 (17.1) | 6753 (28.3) | 6996 (17.2) |

Notes: The healthy diet score, BMI and AMD-PRS were represented as median (IQR), or mean (SD). Other data were n (%). Percentages might not add to 100% due to rounding. BMI=body-mass index. AMD=age-related macular degeneration. PRS=polygenic risk score.

**Supplementary Table 5. Individual components of frailty and their association with AMD incidence**

|  | HR (95% CI) | p value |
| --- | --- | --- |
| Weight loss |  |  |
| Model 1 | 1.15 (1.06 ,1.23) | <0.001 |
| Model 2 | 1.18 (1.09 ,1.27) | <0.001 |
| Model 3 | 1.11 (1.03 ,1.20) | <0.001 |
| Tiredness |  |  |
| Model 1 | 1.03 (0.94 ,1.12) | 0.5545 |
| Model 2 | 1.20 (1.10 ,1.31) | <0.001 |
| Model 3 | 1.13 (1.03 ,1.23) | 0.0078 |
| Low physical activity level | |  |
| Model 1 | 1.23 (1.12 ,1.35) | <0.001 |
| Model 2 | 1.20 (1.09 ,1.32) | <0.001 |
| Model 3 | 1.12 (1.01 ,1.23) | 0.0249 |
| Low grip strength |  |  |
| Model 1 | 1.93 (1.80 ,2.06) | <0.001 |
| Model 2 | 1.30 (1.21 ,1.39) | <0.001 |
| Model 3 | 1.23 (1.14 ,1.32) | <0.001 |
| Low gait speed |  |  |
| Model 1 | 1.86 (1.70 ,2.03) | <0.001 |
| Model 2 | 1.39 (1.27 ,1.52) | <0.001 |
| Model 3 | 1.23 (1.12 ,1.35) | <0.001 |

Notes: The reference group was participants with a normal range for each component. Model 1 was minimally adjusted, which did not adjust any parameter. Model 2 included sociodemographic covariates: age, sex, deprivation, ethnicity, education and income. Model 3 additionally included BMI, lifestyle factors (smoking, drinking, sleep and diet) and diseases status (AMD-RPS, hypertension, diabetes and hyperlipidemia).

**Supplementary Table 6. Association of baseline frailty status with AMD by health status**

|  | Total | Cases | P value | HR (95% CI) | p value for interaction |
| --- | --- | --- | --- | --- | --- |
| **Diseases** |  |  |  |  |  |
| **High blood pressure (HBP)** |  |  |  |  |  |
| Without HBP |  |  |  |  |  |
| Non-frail | 113387 | 1098 |  |  | 0.2205 |
| Pre-frail | 67921 | 943 | <0.001 | 1.27 (1.16 ,1.39) |  |
| Frail | 5350 | 114 | <0.001 | 1.53 (1.25 ,1.87) |  |
| With HBP |  |  |  |  |  |
| Non-frail | 102287 | 1600 |  |  |  |
| Pre-frail | 60219 | 1177 | <0.001 | 1.14 (1.05 ,1.23) |  |
| Frail | 4915 | 131 | <0.001 | 1.39 (1.15 ,1.67) |  |
| **Glucose** |  |  |  |  |  |
| Without high glucose |  |  |  |  |  |
| Non-frail | 183575 | 2252 |  |  | 0.1193 |
| Pre-frail | 106671 | 1691 | <0.001 | 1.18 (1.11 ,1.26) |  |
| Frail | 7994 | 182 | <0.001 | 1.46 (1.25 ,1.71) |  |
| High glucose |  |  |  |  |  |
| Non-frail | 4691 | 90 |  |  |  |
| Pre-frail | 5208 | 155 | 0.01 | 1.46 (1.12 ,1.90) |  |
| Frail | 933 | 35 | 0.01 | 1.68 (1.11 ,2.55) |  |
| **Triglycerides** |  |  |  |  |  |
| Normal triglycerides |  |  |  |  |  |
| Non-frail | 164953 | 2101 |  |  | 0.2965 |
| Pre-frail | 93897 | 1567 | <0.001 | 1.18 (1.11 ,1.27) |  |
| Frail | 6820 | 164 | <0.001 | 1.42 (1.20 ,1.68) |  |
| High triglycerides |  |  |  |  |  |
| Non-frail | 41027 | 466 |  |  |  |
| Pre-frail | 28319 | 448 | 0.01 | 1.20 (1.05 ,1.37) |  |
| Frail | 2908 | 75 | <0.001 | 1.63 (1.26 ,2.11) |  |
| **Hemoglobin concentration** |  |  |  |  |  |
| Normal hemoglobin |  |  |  |  |  |
| Non-frail | 204652 | 2561 |  |  | 0.7048 |
| Pre-frail | 120310 | 1972 | <0.001 | 1.18 (1.11 ,1.25) |  |
| Frail | 9282 | 214 | <0.001 | 1.40 (1.21 ,1.62) |  |
| Low hemoglobin |  |  |  |  |  |
| Non-frail | 5139 | 62 |  |  |  |
| Pre-frail | 4099 | 83 | 0.56 | 1.11 (0.78 ,1.57) |  |
| Frail | 650 | 19 | 0.68 | 0.89 (0.51 ,1.56) |  |
| **Total cholesterol** |  |  |  |  |  |
| Normal total cholesterol |  |  |  |  |  |
| Non-frail | 103914 | 1280 |  |  | 0.1211 |
| Pre-frail | 67551 | 1151 | <0.001 | 1.19 (1.09 ,1.29) |  |
| Frail | 5988 | 144 | <0.001 | 1.33 (1.11 ,1.60) |  |
| High total cholesterol |  |  |  |  |  |
| Non-frail | 102202 | 1289 |  |  |  |
| Pre-frail | 54758 | 867 | <0.001 | 1.17 (1.07 ,1.28) |  |
| Frail | 3749 | 95 | <0.001 | 1.73 (1.39 ,2.14) |  |

Notes: Non-frail participants were used as the reference group. Analyses were adjusted by age, sex, education, ethnicity, deprivation, income, smoking, drinking, sleep, diet, AMD-PRS, hypertension, diabetes and hyperlipidemia, and the corresponding disease was not adjusted in covariate for the subgroup analysis of biochemistry item. HR=hazard ratio.

**Supplementary Figure 1. Non-linear associations between number of individual components of the frailty phenotype and AMD incidence**

Notes: The figure was presented as adjusted HR with the 95% CI shown as shading. Non-frail people were the reference group. Model 1 was minimally adjusted, which did not adjust any parameter. Model 2 included sociodemographic covariates: age, sex, deprivation, ethnicity, education and income. Model 3 additionally included BMI, lifestyle factors (smoking, drinking, sleep and diet) and diseases status (AMD-RPS, hypertension, diabetes and hyperlipidemia). HR=hazard ratio.

**Supplementary Figure 2. Association of baseline frailty status with AMD by sex**

Notes: The subgroup analyzation between gender and BMI was further investigated. Non-frail participants were used as the reference group. Analyses were adjusted by age, sex, education, ethnicity, deprivation, income, smoking, drinking, sleep, diet, AMD-PRS, hypertension, diabetes and hyperlipidemia. HR=hazard ratio.
